# Isolation of two salt-tolerant strains from activated sludge and its COD degradation characteristics from saline organic wastewater

**DOI:** 10.1038/s41598-020-75294-0

**Published:** 2020-10-28

**Authors:** Guizhong Zhou, Xitong Wang, Huiyang Zhao, Weiqian Zhang, Guishan Liu, Xinguo Zhang

**Affiliations:** 1 College of Environmental and Safety Engineering, Qingdao University of Science and Technology, Qingdao, 266042 People’s Republic of China; 2 Environmental Protection Agency, Shandong SilverHawk Chemical Fiber Co. Ltd, Weifang, 261500 People’s Republic of China

**Keywords:** Bioinorganic chemistry, Environmental impact

## Abstract

The efficient biological treatment of saline wastewater has been limited by the low activities of microorganisms under saline conditions. High salinity poses unbalance osmotic stress across the cell wall and even leads to cell plasmolysis. In this work, we aim to isolate salt-tolerant bacterial strains from activated sludge, and apply them for degrading chemical oxygen demand (COD) of saline organic wastewater. Two salt-tolerant strains were screened and isolated from activated sludge, which was domesticated with salty water for over 300 days. The two strains were identified as *Bacillus cereus *(*strain A*) and *Bacillus anthracis *(*strain B*) through 16S rRNA sequencing. The degradation characteristics of strain A were explored. The results showed the relative membrane permeability of *strain A* remained stable under high salt stress, which glycine and proline play an important role to maintain cell osmotic. The protein and soluble sugar amounts of strain were increased by higher salt concentrations. In simulating saline wastewater, the optimum culture temperature, pH, salinity, influent COD concentration and inoculation amount of strain A were 35 °C, 9, 4%, 8000 mg L^−1^, 6%, respectively. Optimal conditions could provide guidance for the treatment of practical saline wastewater. The linear regression model of each impact factor built based on the result PB experiment revealed that cross-linking time has the most significant influence on COD removal for salt-tolerant strains. It will provide theoretical basis for biological treatment of saline organic wastewater.

## Introduction

With the rapid development of industry, agriculture and aquaculture, saline wastewater was consequently produced. Saline wastewater is often considered to be composed of inorganic ions and other contaminants, such as organic matter and heavy metals^1,2^. In general, the mass fraction of total salt is greater than 1%, and the total dissolved solids (TDS) is greater than or equal to 3.5%^3^. The major sources of saline wastewater include agricultural drainage, aquaculture in coastal zones, various industrial sectors, and other secondary sources^4^. Some tannery activities usually produce effluents which contain organic matter, suspended solid (SS), dissolved solids (mainly Cr and acidic ions), ammonia, organic nitrogen, and other specific pollutants^5^. High salinity in effluents could negatively affect microbes, invertebrates, vertebrates and plants^6^ as high salinity could lead to unbalance osmotic stress across the cell wall and even plasmolysis. This would eventually lead to the failure of biological treatment systems^7^.

Saline wastewater is difficult to be treated effectively due to its high salinity and high concentration of COD. Physicochemical, biological, and a combination of ecological engineering processes have been developed for saline wastewater treatment^6^. Physicochemical methods (e.g., adsorption, coagulation-flocculation, advanced oxidation process, membrane separation^8–13^) are effective for COD removal from saline wastewater, whereas the application of these technologies have been limited by the high capital cost and requirements for post treatment of the produed by-prodcuts. The biological reactors contain aerobic methods (e.g., the activated sludge method, sequencing batch reactor), and anaerobic methods (e.g., Anaerobic reactor method). Conventional biological methods cannot be effectively employed due to the inhibited activity of microorganisms in high salinity environment. Therefore, finding salt-tolerant microorganisms becomes a current research hotspot. Salt-tolerant strains, which could efficiently degrade organic matter in saline wastewater, can be isolated from activated sludge domesticated by saline water. The isolation and enrichment of salt-tolerant bacteria from acclimated sludge have been proposed in previous research^14^.

The biological treatment of saline wastewater confronts two major problems, how to improve the removal efficiency of COD in saline organic wastewater, and how to explore the salt tolerance mechanism^3^. It has been shown that the addition of salt-tolerant bacteria restrained the inhibition of salt on microbial activity, and greatly improved the removal of organic matter in wastewater^15^ Several unique adaptations allow halophilic bacteria to survive in high salinity environment. Halophiles accumulate compatible solutes (e.g., K^+^ and amino acid) to equalize the stability balance the high external of cell osmotic pressure. In addition, halophiles have protein and cell wall that contain large numbers of negatively charged amino acids and polar lipids^16^. Ahmadi et al.^17^ used a combination of three salt-tolerant bacteria to treat saline petrochemical wastewater and obtained a COD removal efficiency of 78.7%-61.5%, which shown a decreasing trend along with increasing the organic loading rate. Jiang et al.^14^ screened and acclimated the high-efficiency salt-tolerant colonies to degrade the oil exploitation wastewater. It was showed that the high salinity environment exhibited no obvious inhibitory impact on the salt-tolerant microorganisms, and the COD removal efficiency was about 90% after combined physicochemical process.

The objective of the present study was to isolate salt-tolerant microorganisms from activated sludge and explore its COD degradation characteristics through a series experiments. In this work, two strains of salt-tolerant bacteria were screened from the activated sludge which was domesticated with high salinity wastewater. The salt-tolerant strains were identified and their biodegradation characteristics under high salinity were explored. In addition, conditions for treating salty wastewater were optimized. This study would provide theoretical basis for the treatment of high salinity wastewater, and has important theoretical and practical significance on the field of industry and agriculture.

## Materials and methods

### Bio-containment measures

All of the experiments using screening and cultivating living strains were performed either in a regular Biosafety Level-3 (BSL-3) laboratory.

### Activated sludge and its domestication with saline water

The activated sludge used in this experiment was taken from the return sludge of a sewage treatment plant. The sludge volume index (SVI) was 89.1 mL g^−1^, and the mixed liquid suspended solid (MLSS) was 3.5 g L^−1^. The simulated wastewater consisted of glucose and urea (120:1), with a COD and NH_3_^+^-N concentration of 1200 mg L^−1^ and 200 mg L^−1^, respectively. The concentration of NaCl in the wastewater was controlled at 0–20,000 mg L^−1^ as per experimental requirements.

The sludge and water were mixed 1:1 in this experiment, while the nutrients required for microbial growth were added to the culture medium at 25 °C every 24 h. In 1L sludge solution, follows substances were added: glucose (carbon source) 1 g, ammonium sulfate (nitrogen source) 0.11 g, potassium dihydrogen phosphate (phosphorus source) 0.045 g, which the mass ratio of C: N: P was 100:5:1. In addition, aeration was applied and a dissolved oxygen concentration was maintained at 4 mg L^−1^. In the process of cultivation, sodium chloride was added to the sludge solution to gradually increase the salinity of the system. After about 300 days, the NaCl concentration of the system sludge was gradually increased from 0 to 2% (w/v), and the salt-tolerant sludge was obtained.

### Screening of salt-tolerant strains from activated sludge

The domesticated salt-tolerant activated sludge was applied to a beef extract peptone solid medium with a salinity of 3% (w/v) at 37 °C. The composition for the medium was as follows: 5 g L^−1^ beef extract, 10 g L^−1^ peptone was. Inoculation loops were used, with an aseptic technique to dip into the mixture. The mixed strains were isolated by four zone marking method and cultured in solid medium for 48 h at 37 °C. Single strains grew in the fourth district. Then, six strains capable of growing on solid medium were selected for further isolation and culture until a single colony was isolated and purified. The purified colonies were identified by MIDI-Sherlock Microbial Identification System (MIS).

### Identification of salt-tolerant strains

The isolated and purified strains were subjected to physiological and biochemical experiments such as V-P test and starch hydrolysis test. The genomic DNA of the salt-tolerant bacteria was extracted by the PowerSoil DNA Isolation Kit, and the 16S rDNA samples were directly amplified by polymerase chain reaction (PCR), using universal primers of BSF8/27: 5-AGAGTTTGATCCTGGCTCAG-3 and BSR1510/1492: 5-GGTTACCTTCTTACGACTT-3. Identification at the species level was defined by a 16S rRNA gene sequence similarity of ≥ 99% with the sequence of the type strain in GenBank.

The two identified strains were named strain A and strain B.

### Biodegradation characteristics of the salt-tolerant strains in the saline water

The identified strain A was selected to explore the biodegradation characteristics. Strain A lived in exponential phase was cultured in medium at 35 °C and different kind of ions were added. The concentration of Na^+^ and Cl^−^ in culture medium were controlled in 0.34 mol L^−1^. OD_600_ value of strain A was measured by ultraviolet–visible spectrophotometer after 24 h.

In order to explore the influence of different salt concentrations on the growth of strain A, sodium chloride was added to the medium to gradually increase the salt concentrations of the system. Strain A lived in exponential phase was cultured in 0, 2%, 4%, 6%, 8%, 10% (w/v) salt concentrations medium at 35 °C, respectively. To explore the influence of different compatible substances on growth of strain A, glutamate, glycine, proline, betaine, trehalose were added to medium and concentration of these compatible substances were controlled in 1 g/L. OD_600_ value of strain A was measured by ultraviolet–visible spectrophotometer after 24 h.

Strain A lived in stationary phase was selected to measured intracellular protein and sugar contents under 0, 2%, 4%, 6%, 8%, 10% (w/v) salt concentrations. Strain A lived in exponential phase were selected to measured cell permeability under 0, 2%, 4%, 6%, 8%, 10% (w/v) salt concentrations. In order to confirm the changes of strain’ cell membrane permeability under high salinity, *E.coli* was used as a comparison in this study.

### Optimal conditions of COD degradation in simulating saline organic wastewater by salt-tolerant strains

The removal efficiency of COD in wastewater by salt-tolerant strain A and B under different conditions was investigated by single factor method. These factors were inoculum size, salinity, temperature, pH and COD influent concentration.

Sodium chloride was added to the medium to gradually increase the salt concentrations of the system. Strain A and strain B were immobilized by sodium alginate. Then the PB experiment was designed for the treatment of organic saline wastewater by strain A and B, and the significance of each factor were analyzed by Design-Expert 8.0.6.

### Analysis methods

The concentration of COD was determined by DR1010 laboratory Spectrophotometer (HACH, USA). The OD_600_ absorbance was measured by GS54T type ultraviolet visible spectrophotometer (Shanghai Lengguang Technology Co., Ltd., Shanghai, China). The total intracellular protein was determined by Coomassie brilliant blue staining method^18^. The total amount of soluble sugar in the logarithmic strain was measured by Anthrone method^19^. The relative permeability of cell membranes was determined by Conductivity method^20^. The conductivity was measured by a DDS-11C type conductivity meter (Shanghai Shengke Instrument Equipment Co., Ltd., Shanghai, China). Cell membrane permeability as follows:

$$ {\text{Cell membrane permeability\% }} = \frac{{{\text{L}}_{1} - {\mathrm{Wc}}}}{{{\mathrm{L}}_{0} - {\text{Wc}}}} \times 100{\text{\% }} $$

where L_0_ and L_1_ are conductivity of strains under room temperature (25 °C) and boiled in water bath respectively. W_C_ is the conductivity of deionized water under room temperature (25 °C). In order to confirm the changes of strain’ cell membrane permeability under high salt stress, *E.coli* was used as a comparison in this study. The PB test was designed and analyzed by Design-Expert 8.0.6.

## Results and discussion

### Identification of salt-tolerant strains

After cultured in a medium with 3% (w/v) salinity, the strains with distinct colonies were inoculated into a new medium and cultured at 37° C for 48 h. Two strains were obtained from the salt-tolerant activated sludge after separation and purification, which named strain A and strain B. The morphological description and the physiological and biochemical characteristics of the two strains were shown in Tables 1 and Table 2, respectively.

**Table 1:** Morphology of strain A and strain B.

Strains	Form	Altitude	Wetting degree	Pellucidity	Pigment	Border
A	Circular	Flat	Drier	Opacification	Creamy-white	Regular
B	Circular	Flat	Moist	Opacification	Creamy-white	Irregular

**Table 2:** Physiological and biochemical test results.

Physiological and biochemical indicators	A	B
Gram stain test	+	+
Sugar fermentation test	+	+
Gelatin liquefaction test	−	−
Gelatin liquefaction test	+	−
Urea test	−	−
Citrate test	−	−
Methyl red (M.R) test	−	−
Acetylmethyl alcohol (V–P) test	−	−

An analysis of 16 s RNA sequences of the two strains was performed. The results of the 16sRNA sequence analysis of the two salt-tolerant strains were shown in Tables 3 and Table 4.

**Table 3:** The results of gene sequencing of strain A.

TGGAATTGGGACATGCTATACATGCAGTCGAGCGAATGGATTAAGAGCTTGCTCTTA TGAAGTTAGCGGCGGACGGGTGAGTAACACGTGGGTAACCTGCCCATAAGACTGG GATAACTCCGGGAAACCGGGGCTAATACCGGATAACATTTTGAACCGCATGGTTCG AAATTGAAAGGCGGCTTCGGCTGTCACTTATGGATGGACCCGCGTCGCATTAGCTAG TTGGTGAGGTAACGGCTCACCAAGGCAACGATGCGTAGCCGACCTGAGAGGGTGAT CGGCCACACTGGGACTGAGACACGGCCCAGACTCCTACGGGAGGCAGCAGTAGGG AATCTTCCGCAATGGACGAAAGTCTGACGGAGCAACGCCGCGTGAGTGATGAAGG CTTTCGGGTCGTAAAACTCTGTTGTTAGGGAAGAACAAGTGCTAGTTGAATAAGCT GGCACCTTGACGGTACCTAACCAGAAAGCCACGGCTAACTACGTGCCAGCAGCCGC GGTAATACGTAGGTGGCAAGCGTTATCCGGAATTATTGGGCGTAAAGCGCGCGCAG GTGGTTTCTTAAGTCTGATGTGAAAGCCCACGGCTCAACCGTGGAGGGTCATTGGA AACTGGGAGACTTGAGTGCAGAAGAGGAAAGTGGAATTCCATGTGTAGCGGTGAA ATGCGTAGAGATATGGAGGAACACCAGTGGCGAAGGCGACTTTCTGGTCTGTAACT GACACTGAGGCGCGAAAGCGTGGGGAGCAAACAGGATTAGATACCCTGGTAGTCC ACGCCGTAAACGATGAGTGCTAAGTGTTAGAGGGTTTCCGCCC

**Table 4:** The results of gene sequencing of strain B.

CCACCGACTTCGGGTGTTAAAACTCTCGTGGTGTGACGGGCGGTGTGTACAAGGCC CGGGAACGTATTCACCGCGGCATGCTGATCCGCGATTACTAGCGATTCCAGCTTCAT GTAGGCGAGTTGCAGCCTACAATCCGAACTGAGAACGGTTTTATGAGATTAGCTCCA CCTCGCGGTCTTGCAGCTCTTTGTACCGTCCATTGTAGCACGTGTGTAGCCCAGGTC ATAAGGGGCATGATGATTTGACGTCATCCCCACCTTCCTCCGGTTTGTCACCGGCAG TCACCTTAGAGTGCCCAACTAAATGATGGCAACTAAGATCAAGGGTTGCGCTCGTTG CGGGACTTAACCCAACATCTCACGACACGAGCTGACGACAACCATGCACCACCTGT CACTCTGCTCCCGAAGGAGAAGCCCTATCTCTAGGGTTGTCAGAGGATGTCAAGAC CTGGTAAGGTTCTTCGCGTTGCTTCGAATTAAACCACATGCTCCACCGCTTGTGCGG GCCCCCGTCAATTCCTTTGAGTTTCAGCCTTGCGGCCGTACTCCCCAGGCGGAGTGC TTAATGCGTTAACTTCAGCACTAAAGGGCGGAAACCCTCTAACACTTAGCACTCATC GTTTACGGCGTGGACTACCAGGGTATCTAATCCTGTTTGCTCCCCACGCTTTCGCGCC TCAGTGTCAGTTACAGACCAGAAAGTCGCCTTCGCCACTGGTGTTCCTCCATATCTC TACGCATTTCACCGCTACACATGGAATTCCACTTTCCTCTTCTGCACTCAAGTCTCCC AGTTTCCAATGACCCTCCACGGTTGAGCCGTGGGCTTTCACATCAGACTTAAGAAAC CACCTGCGCGCGCTTTACGCCCAATAATTCCGGATAACGCTTGCCACCTACGTATTAC CGCGGCTGCTGGCACGTAGTTAGCCGTGGCTTTCTGGTTAGGTACCGTCAAGGTGCC AGCTTATTCAACTAGCACTTGTTCTTCCCTAACAACAGAGTTTTACGACCCGAAAGC CTTCATCACTCACGCGGCGTTGCTCCGTCAGACTTTCGTCCATTGCGGAAGATTCCCT ACTGCTGCCTCCCGTAGGAGTCTGGGCCGTGTCTCAGTCCCAGTGTGGCCGATCACC CTCTCAGGTCGGCTACGCATCGTTGCCTTGGTGAGCCGTTACCTCACCAACTAGCTA ATGCGACGCGGGTCCATCCATAAGTGACAGCCGAAGCCGCCTTTCAATTTCGAACCA TGCGGTTCAAAATGTTATCCGGTATTAGCCCCGGTTTCCCGGAGTTATCCCAGTCTTA TGGGCAGGTTACCCACGTGTTACTCACCCGTCCGCCGCTAACTTCATAAGAGCAAGCTC	

The 16S rDNA of strain A and strain B were amplified and sequenced. The sequences of strain A and strain B were imported into the international GenBank database for homology comparison, and were identified as *Bacillus cereus* and *Bacillus anthraci* (with a homology matching indice of 99%) , respectively.

**Figure. 1:**
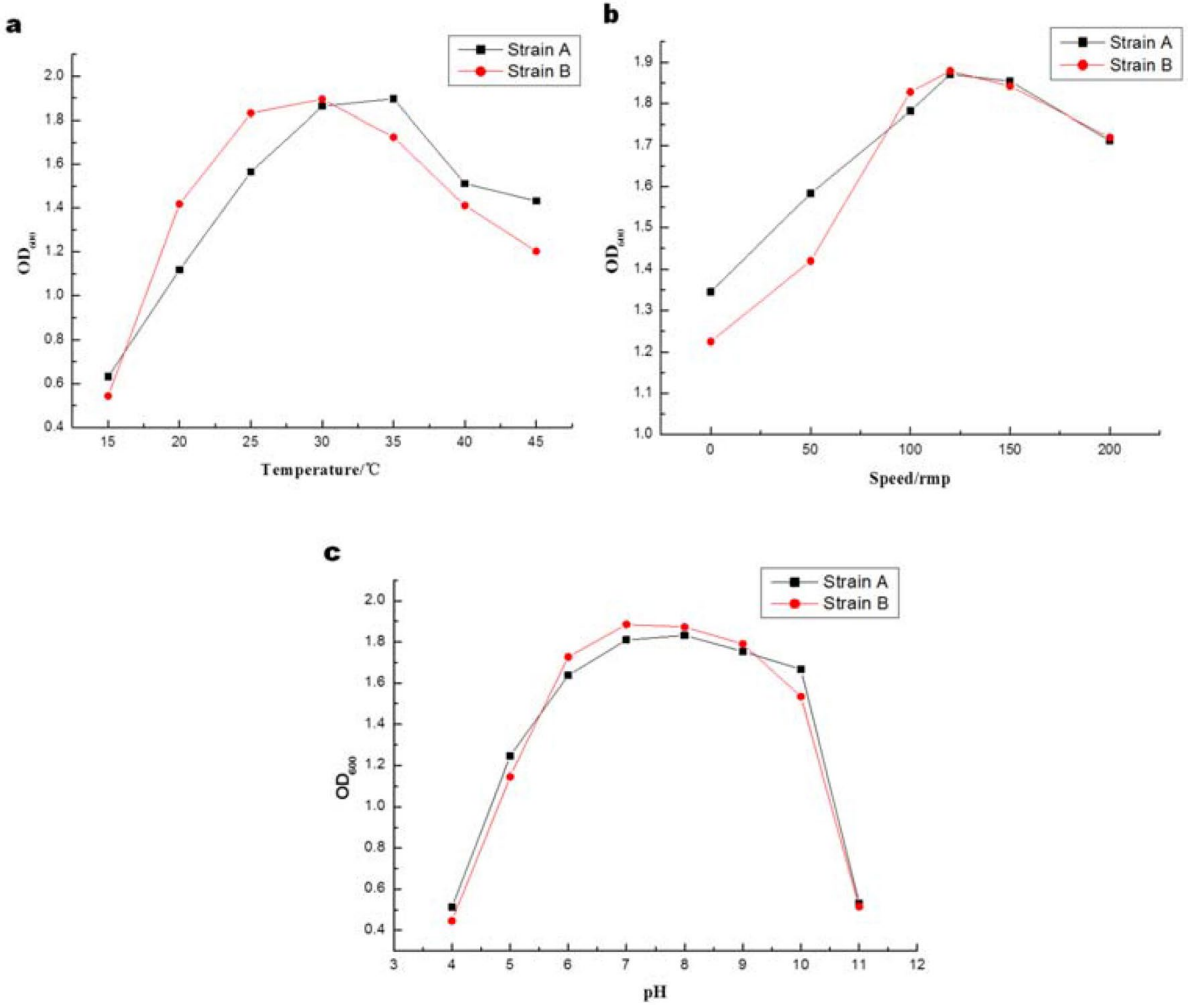
Effects of temperature (**a**), shaking speed (**b**) and pH (**c**) on the growth of the strain.

**Figure. 2:**
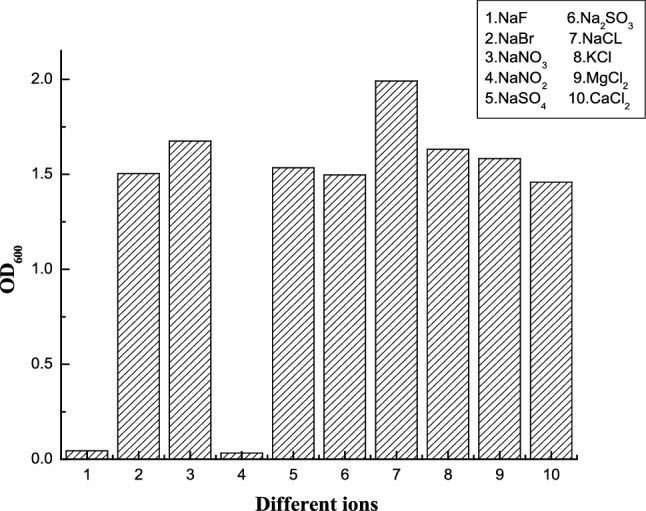
The OD_600_ of strain A under different salts after 24 h cultivation.

The effects of culture temperature, pH and shaking speed on the growth of the strain A and B were explored. The bacterial solution cultured for 24 h was inoculated into the medium, and the absorbance OD_600_ of the culture solution was measured after 24 h of culture under specific conditions. The change curve of bacterial growth under each factor was shown on Fig. 1. Figure 2a was obtained under the pH of 7 and rotation speed of 120 r/min; Fig. 1b was obtained under the temperature of 35 °C and rotation speed of 120 r/min; Fig. 1c was obtained under the pH of 7 and temperature of 35 °C. The results showed that the optimum temperature and pH of strain A was 35 °C, 8.0, respectively. The optimum growth temperature and pH of strain B were 30 °C and 7.0, respectively. In addition, the rotation speed of the shaker also affects the activity of the strain, which the best shaker speed of strain A and B were 120 r min^−1^.

### Biodegradation characteristics of salt-tolerant strain A

In this experiment, strain A (*Bacillus cereus*) after screened and isolation was selected to explore the biodegradation characteristics.

### Effects of different salts on growth of strain A

Different kinds of inorganic salts were added to the culture medium, and the OD_600_ values of strain A cultured in different salts were measured after 24 h cultivation (Fig. 2).

Strain A grew best in NaCl medium and was moderately inhibited in other medium. This means that Cl^−^ has a significant effect on the maintenance of intracellular and extracellular osmotic pressure, and the adaptability of microorganisms will decline when Cl^−^ loss. However, it is not specific because there may be other anion channels in the cell to maintain the osmotic pressure balance. In NaF and NaNO_2_ medium, the growth of the strain was completely inhibited, which indicated that NaF and NaNO_2_ may be toxic to microorganisms. F^−^ and NO_2_^−^ have moderate toxicity, and fluoride could combine with enzymes in cells, and the overall result is the inhibition of bacterial growth^21^. Compared NaCl medium with KCl, MgCl_2_, and CaCl_2_ medium, it was found that microbial growth was inhibited in the last three media, which indicating that the cell’s growth was specifically dependent on Na^+^. There was a sodium ion pump in the cell to maintain the cell material transmembrane transport and osmotic balance, stabilize the structure of the cell membrane and cell wall to ensure the normal metabolism of the microorganisms.

### Effects of different compatible substances on growth of strains A

**Figure. 3:**
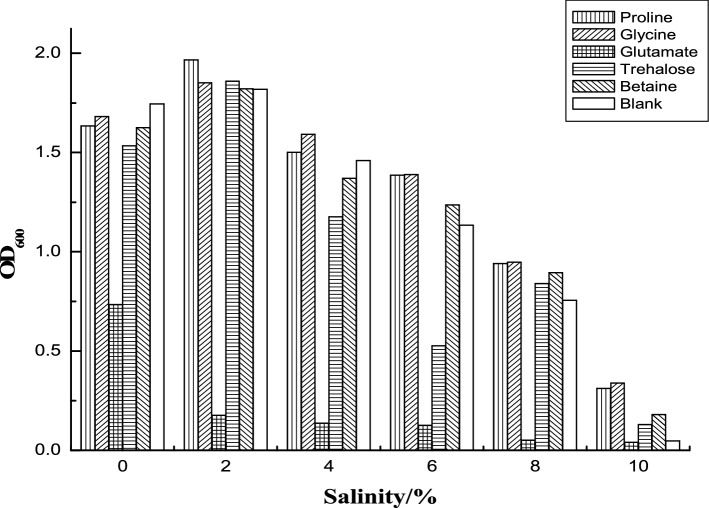
Effects of external addition of compatible solutes on growth of strain A.

Amino acids play an important role in the regulation of solute concentration in salt-tolerant cells. Take the logarithmic growth strain A at 0, 2%, 4%, 6%, 8%, 10% (w/v) salt concentrations, then proline, glycine, glutamic acid, trehalose and betaine with concentration of 1 g L^−1^ were added to explore the effects of different compatible substances on the growth of the strain A. The results were shown in Fig. 3.

As could be seen from the above Fig. 3, the growth of strain A decreased with higher salt concentrations. The difference in the added amino acids also leads to different growth of the strain. Under salt concentrations of 0–4% (w/v), the addition of proline, glycine, trehalose and betaine had no significant effect on the growth of the strain, which was basically the same as that of the blank group. It was reported that proline has a relatively small effect on osmotic balance in halophilic bacteria^22^. Under these conditions, cells can synthesize compatible solutes by itself to maintain osmotic balance, and has no need to uptake these compounds from extracellular environment. Under the condition of 6–10% (w/v) salinity, the addition of the proline, glycine and betaine increased the biomass compared with the blank group. It also showed that when salinity was 10% (w/v), the growth was three times more than that of the blank group. This indicated that the effect of proline and glycine was more evident with high salinity. Proline and glycine are the main compatible solute of Gram-positive bacteria in hypertonic environments^23^. Proline is a major osmoprotectant in certain Gram-positive bacteria, which enhances the cell ability to withstand high osmotic pressure environments^24^.

In fact, the role of compatible solutes goes beyond osmotic adjustment to protect cells and cellular components from freezing, drying, high temperatures and oxygen free radicals, as well as avoiding exposing to carbon sources, nitrogen sources and energy. Therefore, it is common for microorganisms to use a mixture of compatible solutes, a strategy that allows cells to adapt to different environmental damages better^25^.

### Cell membrane permeability of strain A under different salinity

Cell membrane acts as a permeable barrier to the cytoplasm and is able to regulate the transport of macro- and micro- nutrients from the media to the cytoplasm, as well as the osmotic pressure through the plasma membrane^2^. It prevents external substances from entering the cell at will. The permeability of membrane is an important parameter to characterize the damage of cell membrane, which directly reflects whether the cell could maintain normal physiological characteristics.

**Figure. 4:**
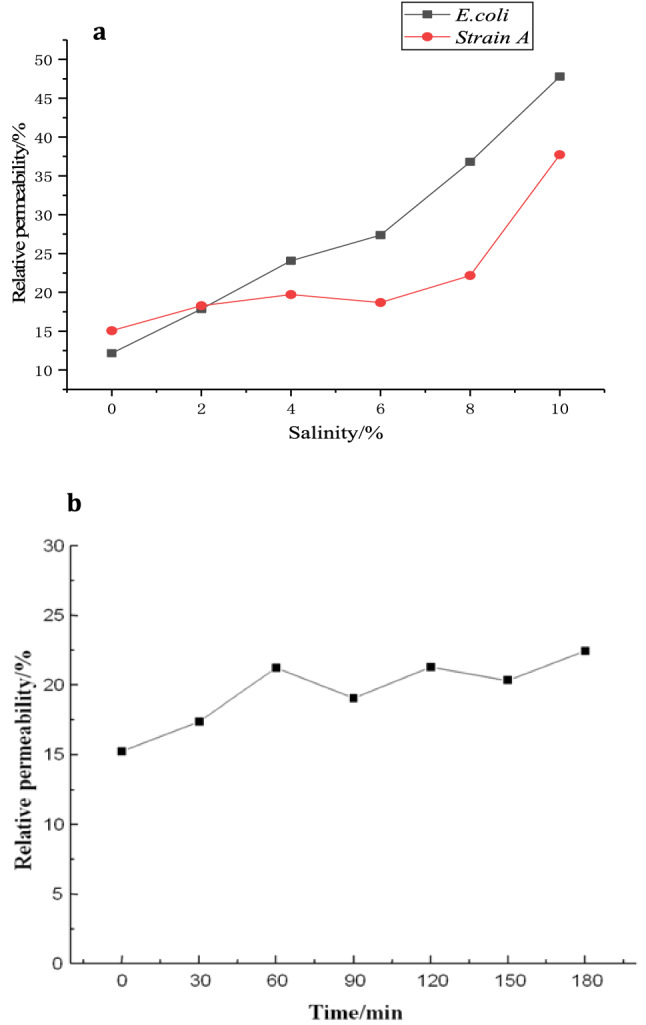
Membrane permeability of strain A and E.coli under different salt concentrations (**a**) and changes in membrane permeability of strain A over time under 8% (w/v) salinity (**b**).

As shown in Fig. 4a, the relative permeability of the strain membrane generally showed an increasing trend with increased salt concentrations. At 0 to 8% (w/v) salt concentrations, membrane permeability was relatively stable. When salinity increased to 10% (w/v), the permeability increased sharply to 43.12%. Cell wall and cell membrane integrity can be destroyed under high salt stress, resulting in the separation of cell wall^26^. In contrast, with the increasing salt concentration, the permeability of *E. coli* membranes increased significantly from 12.5% to 85%, which was much higher than *Bacillus*. Figure 4b shows that the relative permeability of the membrane at 8% (w/v) salinity increased slightly over time. This indicated that the relative permeability of the membrane would not change significantly under certain salt concentration and time frame.

### Total protein content in strain A under different salinities

**Figure. 5:**
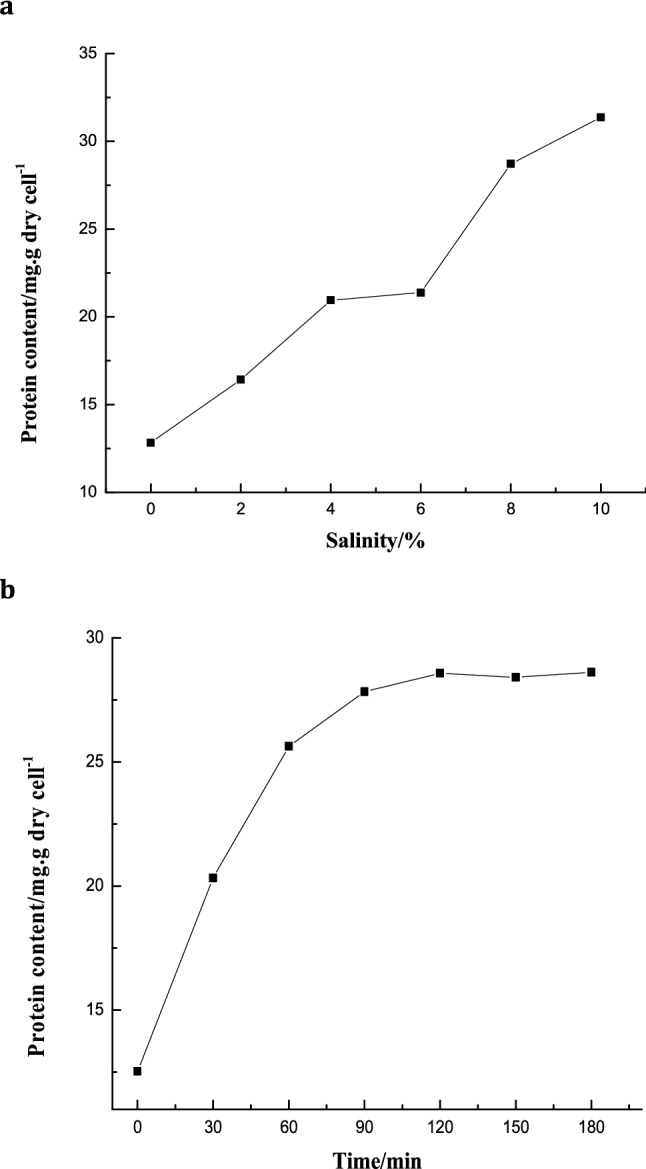
Total protein content in strain A under different salinity (**a**) and changes of total protein content strain A over time under 8% (w/v) salinities (**b**).

As shown in Fig. 5a, the total amount of protein in cells increased along with the increase of salt concentrations, especially under hypertonic conditions. The total intracellular protein of the strain was 15.12 mg∙g dry cell^−1^ under 0% salinity, and it was increased to 31.373 mg∙g dry cell^−1^ when the salinity reached 10% (w/v). It indicated that high salt concentrations could induce the accumulation of macromolecular proteins in the cells of *Bacillus*. The cells accumulated large-scale proteins such as channel proteins and osmotic proteins at high salt conditions. These proteins not only maintain cell osmotic pressure in the high salt extreme environment, but also participate in transporting and synthesizing intracellular material, maintaining amino acid sequences, and ensuring normal cell physiology activity.

Figure 5b shows that under 8% (w/v) salinity, the total amount of protein in the cells increased over time. It increased sharply from 12.533 mg∙g dry cell^−1^ at 0 min to 25.675 mg∙g dry cell^−1^ at 60 min, and then it kept relatively stable at 28.577 mg∙g dry cell^−1^ after 120 min. When subjected to external stress, stain A responded quickly by producing certain proteins to regulate the physiology. This process was in a dynamic equilibrium, where the intracellular protein stopped increased once the equilibrium time was reached. At this time, the cells have adapted to external stress and could metabolized normally under high salt conditions.

### Soluble sugar content in strain A under different salinities

**Figure. 6:**
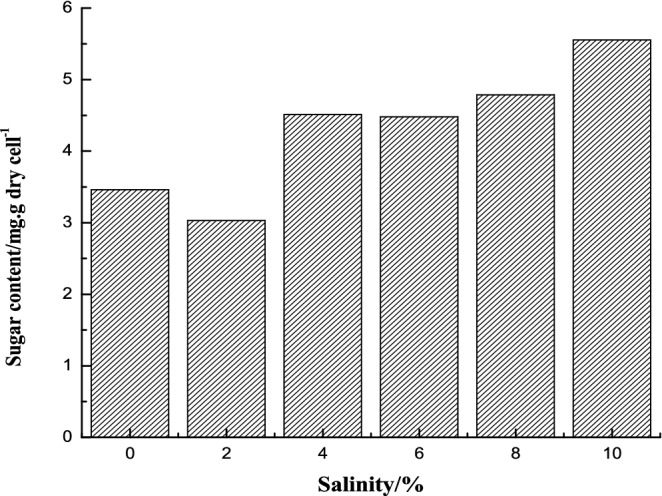
Intracellular soluble sugar content of strain A under different salt concentrations.

Soluble sugars stabilize the protoplast layer on cell membrane surface and protect the intracellular enzyme protein. Strain A at stationary phase was taken to explore the changes of intracellular soluble sugar content under various salinity. The results of intracellular soluble sugar content of strain A under different salt concentrations were shown in Fig. 6. The intracellular soluble sugar content increased with increasing. The intracellular soluble sugar content at 0 salinity was 3.462 mg∙g dry cell^−1^. At 4%, 6%, and 8% (w/v) salinity, the sugar content is roughly equivalent, about 1.3 to 1.4 times than that of 0 salinity, and at the 10% (w/v) salinity the content increased to 1.6 times. It indicated that soluble sugar is not the preferred major infiltration substance for this strain. Lopez et al.^27^ reported that non-salt-tolerant bacteria (which cannot grow in more than 0.5 M NaCl), *E. coli* and *Salmonella*, accumulated soluble sugar trehalose or sucrose as the main osmotic substance. Soluble sugars play an important role as energy suppliers in the process of resisting high salt and high osmotic pressure environment. When strain was stressed by high salt environment, cells need to break down the sugar to release energy. These energy could maintain the synthesis of intracellular macromolecular proteins, nucleic acids and other substances, thus ensuring the normal metabolism, growth and reproduction of the cells.

### Optimal conditions of COD degradation from simulated saline organic wastewater by salt-tolerant strains

#### Optimal cod degradations of saline wastewater by salt-tolerant strains

Simulated organic wastewater was used to explore the effect of systematic salinity, temperature, pH, wastewater COD and inoculation amount on the COD removal efficiency by strain A and B.

**Figure. 7:**
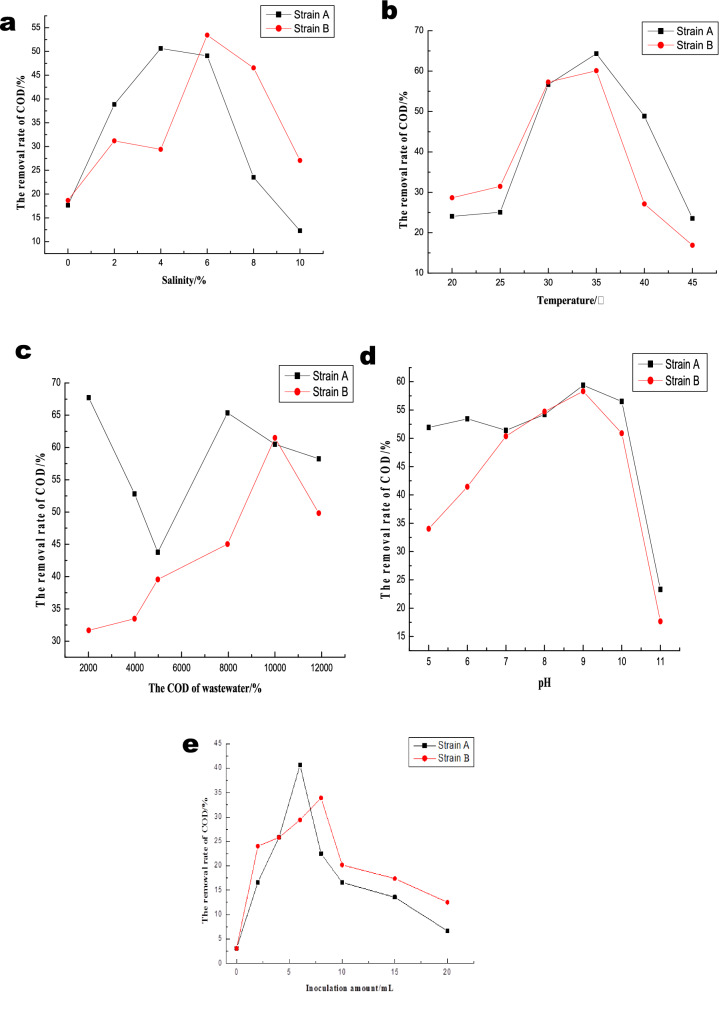
COD removal efficiency of strain A and strain B as affected by different salinity (**a**), external temperature (**b**), wastewater COD concentration (**c**), pH (**d**) and inoculation amount (**e**) after 24 h cultivation.

As shown in Fig. 7, the optimal operational conditions for strain A were as follows: the wastewater influent COD concentration of 8000 mg L^−1^, temperature of 35 °C, pH of 9, salinity of 4% (w/v), and suspension inoculum of 6% (w/v). The optimal treatment conditions for strain B were as follows: the wastewater influent COD concentration of 10,000 mg L^−1^, temperature of 35 °C, pH of 9, salinity of 6% (w/v), and suspension inoculum of 8% (w/v).

### PB test

The strains A and B were immobilized with sodium alginate as embedding agent. Then the PB test design was carried out with immobilized particles A and B to treat organic saline wastewater, and the corresponding PB test design table was obtained.

In Table 5, A, B, C, and D represented the mass concentrations of CaCl_2_, cross-linking time, pH, and addition amount of strains. E, F, G, H, J, K, and L represented virtual variables of software simulation. That means, other variables in the test that may affect the test results. If the dummy variable is a significant factor, it indicates that other key factors are not considered in the test.

**Table 5:** PB test design table of strain A and strain B.

Run	A:CaCl_2_/%	B:Time /h	C:pH	D:V/mL	E	F	G	H	J	K	L	Response of strain A/%	Response of strain B/%
1	3	18	6	4	1	1	− 1	− 1	− 1	1	− 1	50.21	41
2	1	18	8	2	1	1	1	− 1	− 1	− 1	1	44.59	35.24
3	3	6	8	4	− 1	1	1	1	− 1	− 1	− 1	43.56	36.23
4	1	18	6	4	1	− 1	1	1	1	− 1	− 1	49.56	37.83
5	1	6	8	2	1	1	− 1	1	1	1	− 1	40.87	32.54
6	1	6	6	4	− 1	1	1	− 1	1	1	1	42.57	33.28
7	3	6	6	2	1	− 1	1	1	− 1	1	1	41.08	34.45
8	3	18	6	2	− 1	1	− 1	1	1	− 1	1	46.13	37
9	3	18	8	2	− 1	− 1	1	− 1	1	1	− 1	47.07	38.56
10	1	18	8	4	− 1	− 1	− 1	1	− 1	1	1	46.77	38.1
11	3	6	8	4	1	− 1	− 1	− 1	1	− 1	1	43.56	36.23
12	1	6	6	2	− 1	− 1	− 1	− 1	− 1	− 1	− 1	50.21	30.23

**Figure. 8:**
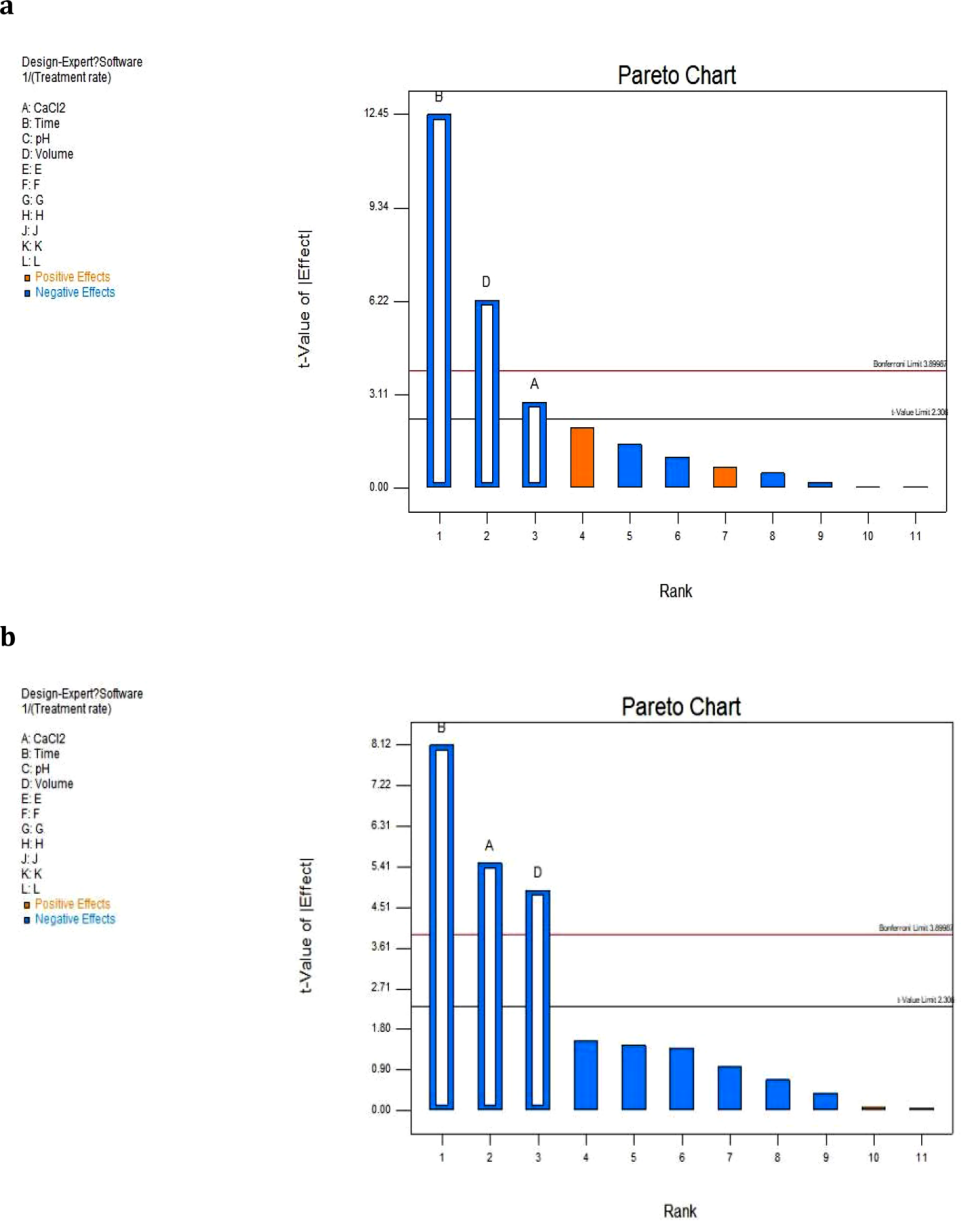
The pareto chart of strain A (**a**) and strain B (**b**). Blue square indicates that factor level is positively correlated with the response value, while orange square indicates that factor level is negatively correlated with the response value.

Pareto charts were designed by Design Expert 8.0.4 software. A pareto chart is a chart arranged in order of importance. As shown in Fig. 8a, the cross-linking time has the greatest influence on strain A, followed by the amount of bacterial liquid embedding, concentration of CaCl_2_, and pH. The cross-linking time contributed the most to the treatment rate, which was 73.72%. Followed by rate of the bacterial liquid embedding amount and concentration of CaCl_2_ were 18.58% and 3.89%, and the pH was less than 0.25%. Figure 8b shows that for the embedded particles B, the cross-linking time has the greatest influence, followed by the concentration of CaCl_2_, the amount of bacterial liquid embedding and the pH. The cross-linking time contributed the most to the treatment rate, which was 51.4596%. Followed concentration of CaCl_2_ and amount of bacterial liquid embedding were 23.6256% and 18.6668%, and the pH was less than 2%.

The regression equation for the treatment rate of each key influencing factor of strain A and stain B could be obtained from the PB test design table, as shown in Eq. (1) and (2) respectively.

1 $$ \frac{1}{R} = 0.027878 + ( - 3.11874 \times 10^{ - 4} A) + ( - 2.26176 \times 10^{ - 4} B) + ( - 6.81217 \times 10^{ - 4} D) $$

2 $$ \frac{1}{R} = 0.036508 + ( - 1.11129 \times 10^{ - 3} A) + ( - 2.73349 \times 10^{ - 4} B) + ( - 9.87852 \times 10^{ - 4} D) $$

According to Table 6 and Table 7, the significant variance P of the two regression equations obtained is less than 0.01%, indicating that the error of this equation is small, and the optimal conditions of the current test could be further calculated according to this model, and could be processed according to the required wastewater. Rate and the range of factors to predict the various conditions required.

**Table 6:** Analysis of variance table of strain A.

Source	Sum of Squares	df	Mean Square	F − value	*p* value	
Model	2.884E−005	3	9.612E−006	67.40	< 0.0001	significant
A-CaCl_2_	1.167E−006	1	1.167E−006	8.18	0.0211	
B-Time	2.210E−005	1	2.210E−005	154.97	< 0.0001	
D-Volume	5.569E−006	1	5.569E−006	39.05	0.0002	
Residual	1.141E−006	8	1.426E−007			
Cor Tota	2.998E−005	11

**Table 7:** Analysis of variance table of strain B.

Source	Sum of Squares	df	Mean Square	F-value	*P* value	
Model	5.881E−005	3	1.960E−005	40.03	< 0.0001	significant
A-CaCl2	1.482E−005	1	1.482E−005	30.26	0.0006	
B-Time	3.228E−005	1	3.228E−005	65.91	< 0.0001	
D-Volume	1.171E−005	1	1.171E−005	23.91	0.0012	
Residual	3.918E−006	8	4.898E−007			
Cor Tota	6.273E−005	11				

According to the model calculation, when the concentration of CaCl_2_ is 2%, the crosslinking time is 18 h, the pH of the embedding agent is 7. When the concentration of the bacterial solution is 2 mL, the immobilized particle A has the best effect on the simulated wastewater. When the CaCl_2_ concentration is 1%, the crosslinking time is 18 h. When the pH value of the burial agent is 7 and the amount of the bacterial liquid is 4 mL, the immobilized granule B has the highest treatment efficiency for the simulated wastewater.

In actual production applications, according to actual needs, in order to achieve a certain processing efficiency, this model could be used to design and select the best solution. Enter the range of values of each factor and the expected processing efficiency. Through this model, multiple processing schemes could be obtained, so that the optimal processing scheme could be selected from the actual operating cost and operating conditions.

## Conclusion

In this work, two salt-tolerant strains were isolated from activated sludge domesticated with saline water. The two salt-tolerant strains were identified as Bacillus cereus and Bacillus anthracis through 16S rRNA sequencing. The biodegradation characteristics of strain A was explored. Proline, glycine and betaine could promote the growth of Bacillus under salinity higher than 6%. The cell membrane of Strain A is relatively well permeable under 0–8% (w/v) salt concentrations, but the cell membrane of the strain was destroyed when the salinity reached 10%( w/v). The cell membrane permeability was significantly increased under 10% salinity, which could severely inhibit strain activity and even lead to cell death. The protein content of Strain A increased with the increase of salinity, and finally reached a dynamic equilibrium. The intracellular soluble sugars of Strain A increased slowly with the increase of salinity. The optimal conditions for high COD removal efficiency from saline wastewater were obtained. And the influence of various factors on COD removal efficiency of salt-tolerant strains was revealed by PB test. It was shown that cross-linking time has the greatest influence on COD removal efficiency for both strains. This work aims to treat the organic wastewater with high salinity by using salt-tolerant strains screened from the activated sludge. We confirm that salt-tolerant strains from activated sludge could effectively degrade COD in saline organic wastewater. The biodegradation characteristics and optimal conditions for saline organic wastewater treatment were revealed, which is of great theoretical and practical significance for biological treatment of saline organic wastewater.

## Supplementary information

Supplementary Information

## Data Availability

The datasets generated during and/or analyzed during the current study are available from the corresponding author on reasonable request.
