# Supplementary material for: Isolation of two salt-tolerant strains from activated sludge and its COD degradation characteristics from saline organic wastewater
Source: Sci Rep. 2020 Oct 28;10:18421. doi: 10.1038/s41598-020-75294-0 (PMC7595179; doi:10.1038/s41598-020-75294-0)
Supplement: Supplementary file 1 — Supplementary Information [file 41598_2020_75294_MOESM1_ESM.docx]

Isolation of two salt-tolerant strains from activated sludge and its COD degradation characteristics from saline organic wastewater

**Guizhong Zhou ^1,*^, Xitong Wang ^1^, , Huiyang Zhao ^1^, ,Weiqian Zhang ^1^, Guishan Liu ^1^, , Xinguo Zhang ^1,2^**

^1^ College of Environmental and Safety Engineering, Qingdao University of Science and Technology, Qingdao 266042, China

^2^ Environmental Protection Agency, Shandong SilverHawk chemical fiber co. LTD, Weifang,

261500, P. R. China.

*** Corresponding authors**. E-mail: zhougz@126.com(G. Zhou)

**Supplementary Table S1** OD_600_ value of strain A and B varied with temperature

| Temperature ℃ | 15 | 20 | 25 | 30 | 35 | 40 | 45 |
| --- | --- | --- | --- | --- | --- | --- | --- |
| OD_600_A | 0.625 | 1.112 | 1.562 | 1.861 | 1.896 | 1.507 | 1.428 |
| OD_600_B | 0.534 | 1.342 | 1.805 | 1.892 | 1.721 | 1.408 | 1.197 |

**Supplementary Table S2** OD_600_ value of strain A and B varied with pH

| pH | 4 | 5 | 6 | 7 | 8 | 9 | 10 | 11 |
| --- | --- | --- | --- | --- | --- | --- | --- | --- |
| OD_600_A | 0.499 | 1.244 | 1.637 | 1.813 | 1.840 | 1.756 | 1.669 | 0.524 |
| OD_600_B | 0.428 | 1.136 | 1.727 | 1.889 | 1.876 | 1.796 | 1.532 | 0.502 |

**Supplementary Table S3** OD_600_ value of strain A and B varied with speed

| Speed r/min | 0 | 50 | 100 | 120 | 150 | 200 |
| --- | --- | --- | --- | --- | --- | --- |
| OD_600_A | 1.345 | 1.581 | 1.782 | 1.867 | 1.854 | 1.707 |
| OD_600_B | 1.221 | 1.418 | 1.824 | 1.877 | 1.842 | 1.718 |

**Supplementary Table S4** The contribution of each factor of strain A to the treatment rate

| **Term** | **Effect** | **SumSqr** | **% Contribution** |
| --- | --- | --- | --- |
| A-CaCl_2_ | -0.000623748 | 1.16718E-006 | 3.89375 |
| B-Time | -0.00271411 | 2.20992E-005 | 73.7233 |
| C-pH | 0.000155799 | 7.282E-008 | 0.242929 |
| D-Volume | -0.00136243 | 5.56867E-006 | 18.5772 |
| E-E | -0.000226322 | 1.53665E-007 | 0.512629 |
| F-F | -1.02287E-005 | 3.13881E-010 | 0.00104711 |
| G-G | -0.000109156 | 3.57448E-008 | 0.119245 |
| H-H | 1.2408E-005 | 4.61874E-010 | 0.00154082 |
| J-J | -0.000317025 | 3.01514E-007 | 1.00586 |
| K-K | -4.10066E-005 | 5.04463E-009 | 0.016829 |
| L-L | 0.00043637 | 5.71257E-007 | 1.91 |

**Supplementary Table S5**  The contribution of each factor of strain B to the treatment rate

| **Term** | **Effect** | **SumSqr** | **% Contribution** |
| --- | --- | --- | --- |
| A-CaCl_2_ | -0.00222258 | 1.48196E-005 | 23.6256 |
| B-Time | -0.00328019 | 3.2279E-005 | 51.4596 |
| C-pH | -0.000585874 | 1.02974E-006 | 1.64163 |
| D-Volume | -0.0019757 | 1.17102E-005 | 18.6686 |
| E-E | -0.000562506 | 9.49239E-007 | 1.51329 |
| F-F | -2.46791E-005 | 1.82718E-009 | 0.0029129 |
| G-G | -0.000275828 | 2.28242E-007 | 0.363867 |
| H-H | -0.00039362 | 4.6481E-007 | 0.741006 |
| J-J | -0.000157823 | 7.47242E-008 | 0.119126 |
| K-K | -0.000623813 | 1.16743E-006 | 1.86113 |
| L-L | 2.62022E-005 | 2.05967E-009 | 0.00328355 |

**Supplementary Figure S1** The results of gene sequencing of strain A

| TGGAATTGGGACATGCTATACATGCAGTCGAGCGAATGGATTAAGAGCTTGCTCTTATGAAGTTAGCGGCGGACGGGTGAGTAACACGTGGGTAACCTGCCCATAAGACTGGGATAACTCCGGGAAACCGGGGCTAATACCGGATAACATTTTGAACCGCATGGTTCGAAATTGAAAGGCGGCTTCGGCTGTCACTTATGGATGGACCCGCGTCGCATTAGCTAGTTGGTGAGGTAACGGCTCACCAAGGCAACGATGCGTAGCCGACCTGAGAGGGTGATCGGCCACACTGGGACTGAGACACGGCCCAGACTCCTACGGGAGGCAGCAGTAGGGAATCTTCCGCAATGGACGAAAGTCTGACGGAGCAACGCCGCGTGAGTGATGAAGGCTTTCGGGTCGTAAAACTCTGTTGTTAGGGAAGAACAAGTGCTAGTTGAATAAGCTGGCACCTTGACGGTACCTAACCAGAAAGCCACGGCTAACTACGTGCCAGCAGCCGCGGTAATACGTAGGTGGCAAGCGTTATCCGGAATTATTGGGCGTAAAGCGCGCGCAGGTGGTTTCTTAAGTCTGATGTGAAAGCCCACGGCTCAACCGTGGAGGGTCATTGGAAACTGGGAGACTTGAGTGCAGAAGAGGAAAGTGGAATTCCATGTGTAGCGGTGAAATGCGTAGAGATATGGAGGAACACCAGTGGCGAAGGCGACTTTCTGGTCTGTAACTGACACTGAGGCGCGAAAGCGTGGGGAGCAAACAGGATTAGATACCCTGGTAGTCCACGCCGTAAACGATGAGTGCTAAGTGTTAGAGGGTTTCCGCCC |
| --- |

**Supplementary Figure S2** The results of gene sequencing of strain B

| CCACCGACTTCGGGTGTTAAAACTCTCGTGGTGTGACGGGCGGTGTGTACAAGGCCCGGGAACGTATTCACCGCGGCATGCTGATCCGCGATTACTAGCGATTCCAGCTTCATGTAGGCGAGTTGCAGCCTACAATCCGAACTGAGAACGGTTTTATGAGATTAGCTCCACCTCGCGGTCTTGCAGCTCTTTGTACCGTCCATTGTAGCACGTGTGTAGCCCAGGTCATAAGGGGCATGATGATTTGACGTCATCCCCACCTTCCTCCGGTTTGTCACCGGCAGTCACCTTAGAGTGCCCAACTAAATGATGGCAACTAAGATCAAGGGTTGCGCTCGTTGCGGGACTTAACCCAACATCTCACGACACGAGCTGACGACAACCATGCACCACCTGTCACTCTGCTCCCGAAGGAGAAGCCCTATCTCTAGGGTTGTCAGAGGATGTCAAGACCTGGTAAGGTTCTTCGCGTTGCTTCGAATTAAACCACATGCTCCACCGCTTGTGCGGGCCCCCGTCAATTCCTTTGAGTTTCAGCCTTGCGGCCGTACTCCCCAGGCGGAGTGCTTAATGCGTTAACTTCAGCACTAAAGGGCGGAAACCCTCTAACACTTAGCACTCATCGTTTACGGCGTGGACTACCAGGGTATCTAATCCTGTTTGCTCCCCACGCTTTCGCGCCTCAGTGTCAGTTACAGACCAGAAAGTCGCCTTCGCCACTGGTGTTCCTCCATATCTCTACGCATTTCACCGCTACACATGGAATTCCACTTTCCTCTTCTGCACTCAAGTCTCCCAGTTTCCAATGACCCTCCACGGTTGAGCCGTGGGCTTTCACATCAGACTTAAGAAACCACCTGCGCGCGCTTTACGCCCAATAATTCCGGATAACGCTTGCCACCTACGTATTACCGCGGCTGCTGGCACGTAGTTAGCCGTGGCTTTCTGGTTAGGTACCGTCAAGGTGCCAGCTTATTCAACTAGCACTTGTTCTTCCCTAACAACAGAGTTTTACGACCCGAAAGCCTTCATCACTCACGCGGCGTTGCTCCGTCAGACTTTCGTCCATTGCGGAAGATTCCCTACTGCTGCCTCCCGTAGGAGTCTGGGCCGTGTCTCAGTCCCAGTGTGGCCGATCACCCTCTCAGGTCGGCTACGCATCGTTGCCTTGGTGAGCCGTTACCTCACCAACTAGCTAATGCGACGCGGGTCCATCCATAAGTGACAGCCGAAGCCGCCTTTCAATTTCGAACCATGCGGTTCAAAATGTTATCCGGTATTAGCCCCGGTTTCCCGGAGTTATCCCAGTCTTATGGGCAGGTTACCCACGTGTTACTCACCCGTCCGCCGCTAACTTCATAAGAGCAAGCTC |
| --- |
